# SimB16: Modeling Induced Immune System Response against B16-Melanoma

**DOI:** 10.1371/journal.pone.0026523

**Published:** 2011-10-19

**Authors:** Francesco Pappalardo, Ivan Martinez Forero, Marzio Pennisi, Asis Palazon, Ignacio Melero, Santo Motta

**Affiliations:** 1 University of Catania, Catania, Italy; 2 CIMA and CUN University of Navarra Pamplona, Pamplona, Spain; Dana-Farber Cancer Institute, United States of America

## Abstract

Immunological therapy of progressive tumors requires not only activation and expansion of tumor specific cytotoxic T lymphocytes (CTLs), but also an efficient effector phase including migration of CTLs in the tumor tissue followed by conjugation and killing of target cells. We report the application of an agent-based model to recapitulate both the effect of a specific immunotherapy strategy against B16-melanoma in mice and the tumor progression in a generic tissue section. A comparison of the *in silico* results with the *in vivo* experiments shows excellent agreement. We therefore use the model to predict a critical role for CD137 expression on tumor vessel endothelium for successful therapy and other mechanistic aspects. Experimental results are fully compatible with the model predictions. The biologically oriented *in silico* model derived in this work will be used to predict treatment failure or success in other pre-clinical conditions eventually leading new promising *in vivo* experiments.

## Introduction

Despite intensive research, cancer is still a leading cause of death worldwide. Immunotherapy is a promising therapeutic strategy for different types of cancer, but in its present form it is often not sufficient to control tumor growth in patients. Antigen specific cytotoxic T lymphocytes represent a crucial component of the adaptive immune system with particular importance in the eradication of intracellular pathogens and malignant cells[1]. Considering their prominent role in cellular immunity, there is a comprehensible interest in targeting cytotoxic T-lymphocytes to cancer. But even so adoptively transferred CTLs can be observed infiltrating the tumor, their capacity to control tumor growth is still insufficient in most patients[2]. Malignancies actively shield themselves from immune attack. Soluble and membrane-attached molecules whose normal function is to regulate immunity and avoid self reactivity are cunningly perverted by tumors to permit immune escape[3].

Tumor-infiltrating lymphocytes are rendered anergic through the actions of co-inhibitory molecules expressed on the surface of tumor and stroma cells. Successful immunotherapy requires combined strategies that are able to turn-off deleterious signals while enhancing CTLs migration and overall killing capacity[4]–[6].

CD137, also known as 4-1BB, is a co-stimulatory protein expressed on activated T, NK, B-lymphocytes, dendritic cells and tumor endothelium[7], [8]. CD137 natural ligand, CD137L, is present on the surface of activated antigen presenting cells[9]. Artificial stimulation of this molecule with monoclonal antibodies therapeutically augments the cellular immune response against tumors[10]. The mechanism of action is multilayered and includes effects on both immune and non-immune cells (i.e. endothelial cells). These powerful agents can be combined with adoptive T cell lymphocytes as well as chemotherapy and other immuno-modulating agents to achieve an enhanced anti-tumoral activity [5], [11]–[13] and avoid undesired side effects like the hepatotoxicity that has been recently described under anti-CD137 high dosage treatment[14].

CTLs migration to tumor site is a key limiting factor to adoptive cell therapy efficacy. We have recently shown that CD137 is expressed on tumor endothelial cells. Ligation of CD137 on tumor endothelium unleashes a pro-inflammatory switch that promotes the entry of CTLs inside the tumor[7]. In this regard, CD137 monoclonal antibody (mAb) enhances T cell migration and cytotoxic activity.

The agent based model derived in this work is first tuned to reproduce the available *in vivo* results. After this tuning phase it is used to predict the role of CD137 on endothelial cells. This is an important step in the area of melanoma treatment as currently there is no animal model available to fully isolate the role of CD137 on endothelial cells in tumor rejection after immunotherapy.

## Results and Discussion

We analyzed six pre-clinical cases of B16-OVA melanoma treatment in immunocompetent mice, namely: *i)* **Control**, i.e. mice that received no treatment; *ii)* **Anti-CD137**, i.e. mice that received i.p. 100 ug of anti-CD137 monoclonal antibody; *iii)* **Non activated OT-1**, i.e. mice treated with 2×10^6^ antigen naïve OT-1-T cells i.v.; *iv)* **Anti-CD137+Non activated OT-1**, i.e. mice that got i.p. 100 ug of anti-CD137 monoclonal antibody and i.v. 2×10^6^ naïve OT-1-T cells; *v)* **Activated OT1**, i.e. mice that received 2×10^6^ antigen activated OT-1-T cells i.v.; *vi)* **Anti-CD137+Activated OT-1**, i.e. mice that got i.p. 100 ug of anti-CD137 monoclonal antibody and i.v. 2×10^6^ activated OT-1-T cells.

We performed 100 *in silico* experiments for each of the five treated cases plus 100 for the untreated case. Time zero of the simulation corresponds to a 6–8 weeks old mouse. The simulation ends at day 33 post-tumor injection.

**Figure 1:**
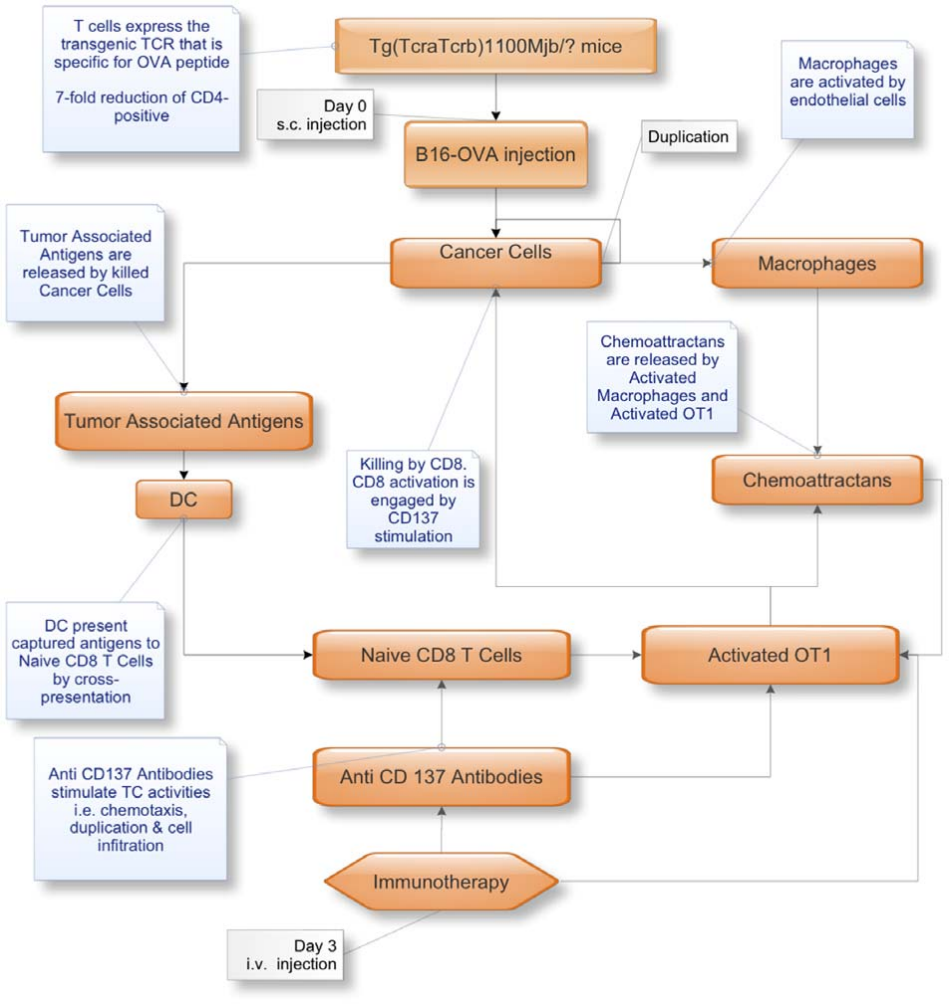
The conceptual model. This figure conceptually explains the biological workflow. It represents the first step for successfully modeling the scenario. Arrows represent the logical flow. Labels explain the interactions or the actions (i.e., status change, activities and functions) by the involved entities.

**Figure 2:**
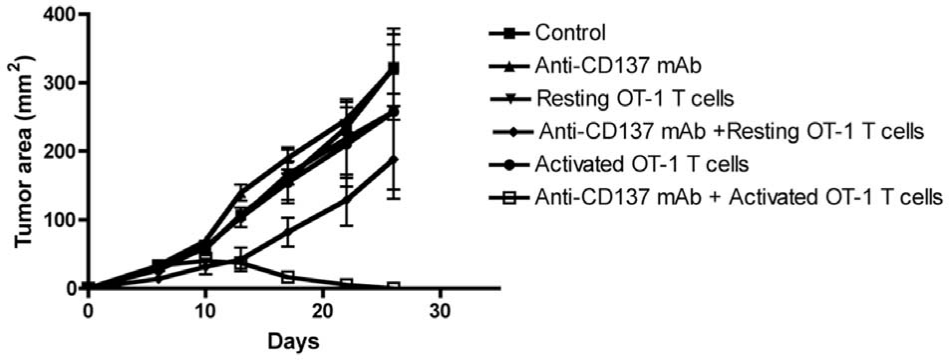
Activated OT-1 cells and systemic injection of anti-CD137 mAb show therapeutic synergy against B16-OVA melanoma. Mice were subcutaneously implanted with B16-OVA cells on day 0 and treated on day 3 with 100 µg of rat IgG (control) or anti-CD137 mAb i.p. Mice also received on the same day activated or resting OT-1 cells i.v.

Figure 1 and Figure 2 show the tumor growth for the six cases in the *in vivo* and in the *in silico* experiments, respectively.

A comparison with Figure 1 shows excellent agreement with *in vivo* experiments. Substantially, in the *in vivo* experiments we don't observe any significant difference among control, treated with Anti-CD137 mAb and treated with non activated OT-1 mice: the tumor growth is not affected at all. Looking at the correspondent curves in Figure 2, we find the same tumor growth dynamics. Looking at the cases of mice treated with activated OT-1 T cells and resting OT-1 + Anti-CD137 we observe a light reduction of the tumor area (as reported perfectly also in the *in silico* curves), but the tumor growth velocities are not affected. Finally in the mice treated with activated OT-1 T cells + Anti-CD137 mAb the tumor is almost totally rejected by day 20. Even in this case, simulations show an excellent agreement with *in vivo* data.

**Figure 3:**
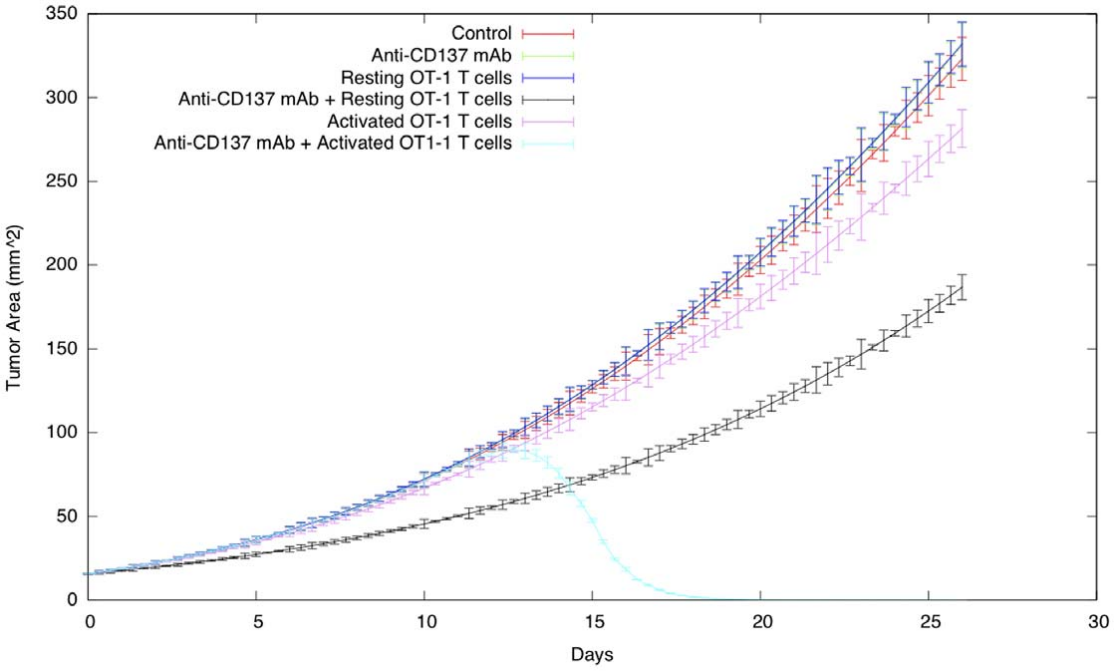
Tumor-area curves of virtual mice receiving OT-1 T cells and anti-CD137 mAb. B16-OVA cells were injected toward the immediate neighborhood in the center of lattice at timestep 0. Immunotherapy on day 3 with 100 µg of rat IgG (control) or anti-CD137 mAb i.p. were simulated injecting the compound around the lattice walls. Virtual mice that also received in the same day activated or resting OT-1 T cells i.v. were simulated in the same way.

**Figure 4:**
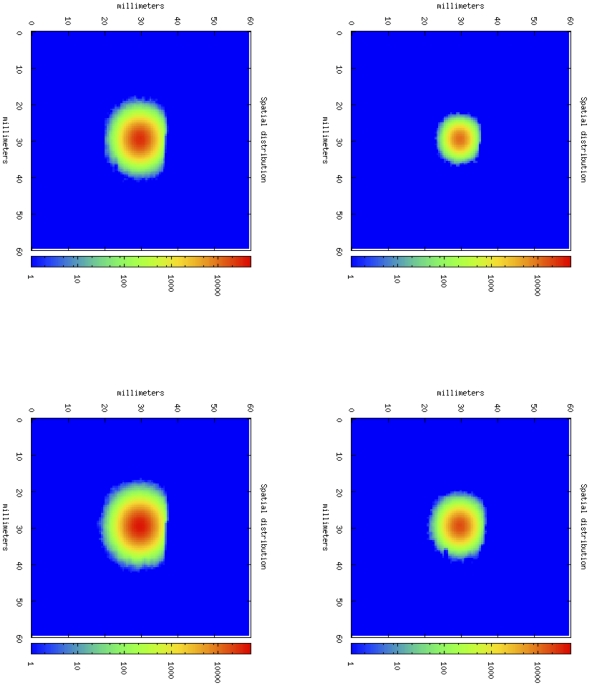
In silico tumor growth dynamics in a typical simulation of an untreated mouse. From upper-left, following clock-wise way, cancer cells dynamics at day 7, 14, 21, 28 following tumor cell inoculation.

**Figure 5:**
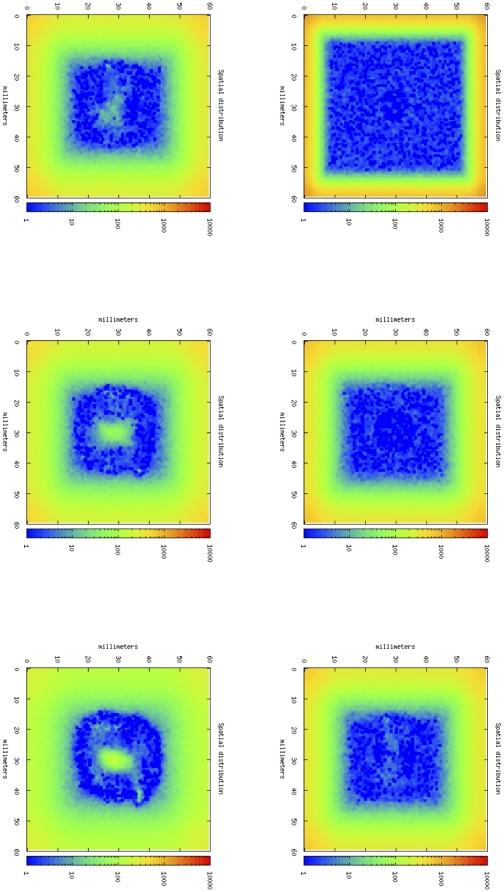
Cytotoxic T cells dynamics. With the anti-CD137 stimulation, TC cells are able to infiltrate tumor and consequently they kill cancer cells, causing total rejection of B16- melanoma. From upper-left, following clock-wise way, OT-1 cells dynamics at day 7, 14, 16, 20, 24, 28.

**Figure 6:**
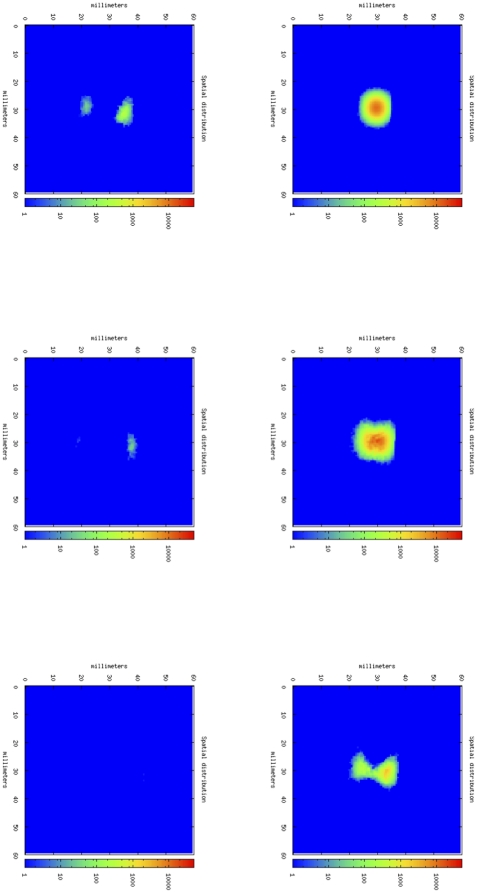
In silico tumor growth dynamics in a typical simulation of a mouse treated with activated OT-1 cells and anti-CD137. From upper-left, following clock-wise way, OT-1 cells dynamics at day 7, 14, 16, 20, 24, 28.

Figure 3 shows four phases of tumor growth dynamics in a typical simulation of an untreated mouse. It depicts the tumor growth as spatial distribution of cancer cells in the simulated portion of mouse tissue. In this case, TC cells are almost unable to infiltrate into the core of the tumor. If we compare this situation with the one showed by Figure 4, we note that in this case, thanks to the effect of anti-CD137 mAb, OT-1 T cells are able to infiltrate the tumor core, and finally destroy cancer cells (Figure 5). The simulation is then able to capture the major effect of anti-CD137, i.e. the augment of the cytotoxicity of TC cells and the ability to follow chemotaxis gradients and consequently to infiltrate the tumor (Figure 6).

**Figure 7:**
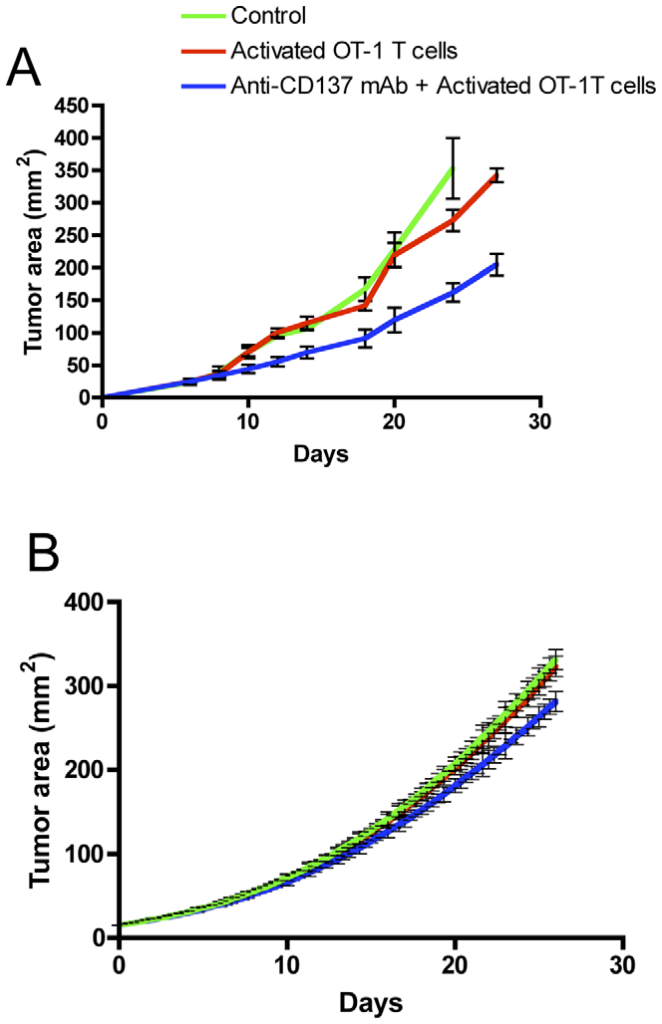
Combined treatment started at day 8 is not effective to eliminate B!6 melanoma. **A. In vivo experiment.** Mice were subcutaneously implanted with B16-OVA cells on day 0 and treated on day 8 with 100 µg of rat IgG (control) or anti-CD137 mAb i.p. Mice also received on the same day activated or resting OT-1 cells i.v. **B. In silico experiment.** B16-OVA cells were injected toward the immediate neighborhood in the center of lattice at timestep 0. Immunotherapy on day 8 with 100 µg of rat IgG (control) or anti-CD137 mAb i.p. were simulated injecting the compound around the lattice walls. Virtual mice that also received in the same day activated or resting OT-1 T cells i.v. were simulated in the same way.

In a different experiment, we started treatment with OT-1 activated T cells or OT-1 activated T cells + Anti-CD137 mAb at day 8 after tumor inoculation. Figure 7A displays the results for the *in vivo* experiment. When treatment is initiated at the indicated time, the combined therapy is completely ineffective, suggesting a relatively reduced tumor T cell infiltration capable of eradicating B16-OVA melanoma. *In silico* experiments nicely reproduce the *in vivo* experimental results. Our computational model is able to capture the underlying dynamics of combined immunotherapy in two different experimental scenarios that include 9 different treatment options.

### Model predictions

**Figure 8:**
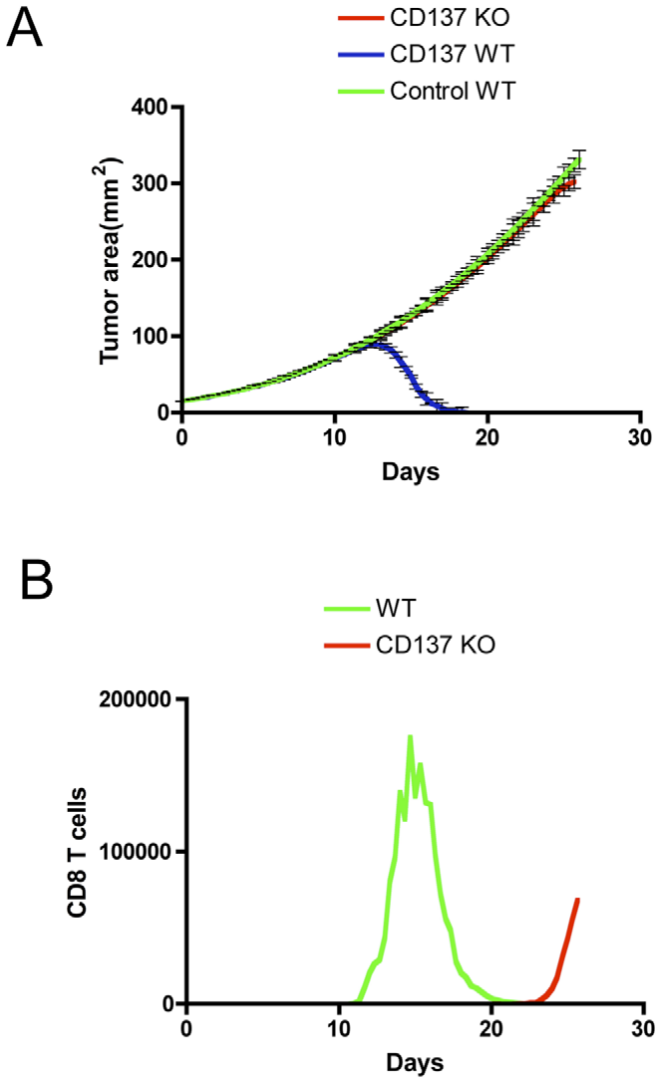
Model prediction. CD137 expression on endothelial cells is essential for tumor rejection. **A. In silico experiment.** WT or CD137 KO (only on endothelial cells) virtual mice were treated as in Figure 3. **B.** CD8 T cell infiltration after combined therapy in WT and or CD137 KO virtual mice.

We have previously shown that CD137 is expressed on tumor endothelial cells[7]. CD137 activation with agonist monoclonal antibodies on tumor endothelial cells up regulates adhesion molecules and promotes T cell infiltration inside tumors. Currently, there is no animal model available to fully isolate the role of CD137 on endothelial cells in tumor rejection after immunotherapy. Such animal model, CD137 conditional knockout on endothelial cells can be simulated using the agent based model described in this work. Combined immunotherapy under the same conditions as described in Figure 3 does not eliminate B16-OVA in mice that do not express CD137 on endothelium (Figure 8A). CD8 T cell infiltration is drastically reduced and delayed in time compared to wild type in silico mice (Figure 8B). Entrance of killer T cells at late time points is ineffective to eliminate a tumor burden beyond 1 cm in diameter. Therefore early infiltration of T cells that seems to be dependent on CD137 expression on tumor vasculature is predicted as an important factor to dictate the therapeutic results. Although the predictions for endothelium CD137 involvement in therapy are of much interest, clearly demand confirmatory experimental verification. However, previous experimental results are fully compatible with this hypothesis[7], [8], [22].

For cost-effectiveness analysis one can estimate that each group of six mice (as those shown in the present paper) developing tumors and being treated takes around 600-800 $ taking into account: per mouse cost, animal facility personnel, investigator time, treatment cost (T cells+antibodies). Typical experiments would cost about 1,800–2000$. Having this estimation in mind, it is clear that an in silico approach can save both money and time, suggesting target experiments and then minimizing the number of needed experiments.

Biological data availability has grown enormously in the past two decades. Such a large amount of data requires a computational and logical framework to be organized and analyzed.

In this scenario, computational models are powerful tools for knowledge discovery both for biological and medical sciences. To achieve this goal, one needs both descriptive and predictive computational models. A descriptive model reproduces *in silico* results obtained from wet laboratory experiments or clinical treatment. If a predictive model is able to forecast the outcome of new possible experiments or treatments and this model capability is verified by a new experiment or independent clinical data, then it can be used as predictive model. Then the model can be used to forecast *in silico*, with much less cost and effort, the outcome of many bio-experiments or clinical trials and rank them according to the quality of their results. Only the best experimental condition will be tested *in vivo* saving money, time and animal distress. As it is well known, models improve in the virtuous cycle of experiment – model – experiment widely used in the physical sciences.

In this paper we presented a model to analyze the co-stimulatory effect of anti-CD137 mAb for the melanoma treatment upon synergistic adoptive transfer of activated OT-1 T cells. The reported *in vivo* show that a single administration of anti-CD137 mAb plus activated OT-1 T cells is sufficient to completely reject the B16-OVA, while single components or not activated OT-1 T cells have no success.

The *in silico* experiments performed with the presented computational model show very good agreement with their *in vivo* counterpart. The model has been then used to identify the role of CD137 on endothelial cells in tumor rejection after immunotherapy, as currently there is no animal model to conduct these kind of experiments. The model predicts that early infiltration of T cells seems to be dependent on CD137 expression on tumor vasculature. These predictions clearly demand confirmatory experimental verification; however, previous experimental results are fully compatible with this hypothesis.

Many aspects of CD137 molecule biology are still not fully understood. Investigating these aspects requires many difficult and expensive wet lab experiments. Our ongoing goal is to test the predictive ability of the model with few illustrating scenarios and then use the model as a virtual lab to analyze different aspects and hypotheses regarding this mode of immunotherapy. This work is still in progress and results will be published in due course.

## Materials and Methods

The biological scenario in which the B16-OVA melanoma and the related induced immune response operate, need to be described in a rationale manner, before they can be translated into a mathematical/computational description. This means that the description must be able to capture the essential properties of the phenomenon i.e., the system entities, their organization and their dynamic behavior.

The role of a conceptual model is then to drive biological knowledge into a solvable mathematical representation, offering a conceptual framework for iterative thinking about the scientific domain allowing the inclusion of additional properties in the same scheme with a limited effort.

To satisfy the above properties, the first task in building a solid conceptual model is to identify all the relevant entities and their properties (cells, molecules, adjuvants, cytokines, interactions) that biologists and clinicians recognize as crucial in the *in vivo* scenario. Next, the modeler has to categorize all the interactions among entities that play a relevant role. These must be described using biological knowledge inside a logical framework, to be able, as a further step, to map them from the biological world to a mathematical/computational one. The final step is to set all the biological constants, relevant functions, and identify the best computational framework capable of hosting the simulated biological scenario.

Having all the above described steps in mind, the first task to deal with is the choice of the entities we have to include in the model. We considered both cellular and molecular entities.

We considered both B lymphocytes and T lymphocytes. Plasma B cells (P) were inserted as specific antibodies producers. Both cytotoxic T cells (TCs) and helper T cells (TH) were inserted in the model. Monocytes are represented as well and we take care of macrophages (M). Dendritic cells play the role of cross-presentation antigen processing cells. Two major types of dendritic cells have been discovered and have been named conventional (cDCs) and plasmacytoid dendritic cells (pDCs). pDCs are not able to present the antigen, so we represented only cDCs in the model, along with their specific capability of processing and cross-presenting antigen peptides both in MHC class I and MHC class II molecules. Immune complexes (ICs) were also inserted in the model and treated as antibody-tumor antigen complex[15].

Several molecules entities engage an important role in simulating the B16-OVA melanoma biological scenario. The model distinguishes between simple small molecules like interleukins or signaling molecules in general and more complex molecules like immunoglobulins and antigens, for which we need to represent the specificity. Regarding the interleukin class molecules, we represent interleukin 2 (IL-2) that is necessary for the development of T cell immunologic memory, one of the unique characteristics of the immune system, which depends upon the expansion of the number and function of antigen-selected T cell clones.

Immunoglobulins of class IgG are represented as well. There is no immunoglobulins class switching representation, because we don't need to represent other classes of Ig and because IgG is the most versatile immunoglobulin since it is capable of carrying out all of the functions of immunoglobulins molecules. Moreover IgG is the major immunoglobulin in serum (75% of serum Ig is IgG) and IgG is the major Ig in extra vascular spaces.

The model also contains dominant characteristics of the OT-1 transgenic mice that were used in *in vivo* experiments, i.e.:

- all CD8 single positive T cells in the thymus express the transgenic TCR that is specific for OVA peptide in the context of H2-Kb;
- the CD8 single positive T cells in the thymus express high levels of the transgenic TCR;
- increased CD8-positive T cell number respect to CD4-positive (7-fold reduction of CD4-positive)
- CD8 T cells have high cytolytic activity toward target cells containing OVA peptide.

A detailed overview of the conceptual model is shown in Fig. 1.

The biological dynamics of the cells is realized by state-changes: each cellular entity is labeled by a suitable state that describes its current biological condition (naïve, activated, duplicating and so on). The state can change when a cell interacts with another cell, with a molecule or with both of them.

Next step is to identify the relevant interactions that happen during the induced immune response against B16 melanoma. We included the following interactions.

- *B lymphocyte and helper T lymphocyte interaction*. If the T receptor (CD4) at the surface of a T helper lymphocyte binds specifically peptide/major histocompatibility complex class II at the surface of the antigen presenting B lymphocyte, helper T lymphocyte proliferates and secretes interleukin 2. At the same time, B lymphocyte proliferates and differentiates into a plasma cell.
- *Macrophage and helper T lymphocyte interaction*. If a T cell receptor (CD4) at the surface of T helper lymphocyte binds specifically peptide/major histocompatibility complex class II at the surface of antigen processing macrophage cell, helper T lymphocyte proliferates and secretes interleukin 2.
- *Dendritic cell with B16 melanoma tumor associated antigen*. If a cDC encounters a B16 melanoma tumor associated antigen, the cDC internalizes the antigen and processes it into peptides that are then presented by major histocompatibility complex class II or by major histocompatibility complex class I (cross-presentation ability of cDCs) at the dendritic cell surface. cDC becomes antigen presenting cell.
- *Cytotoxic T cell with antigen presenting cDC interaction*. If a T cell receptor (CD8) at the surface of cytotoxic T lymphocyte binds specifically peptide/major histocompatibility complex class I at the surface of antigen processing dendritic cell, cytotoxic T lymphocyte begins activated (primed).
- *Cytotoxic T cell with B16-OVA melanoma cell interaction*. If the T cell receptor at the surface of an activated T cytotoxic lymphocyte binds specifically peptide/major histocompatibility class I at the surface of B16-OVA melanoma cell, in presence of IL-2 and anti-CD137 the TC kills B16-OVA melanoma cell.
- *Anti*-*CD137 with activated cytotoxic T cell.* Anti-CD137 will improve cytotoxicity, duplication rate and chemotaxis sensitivity of activated cytotoxic T cell.

The model takes into consideration a 2D domain physical space with periodic boundaries. Apparently this seems a limitation in terms of space representation. On the other hand we know that all the processes and interactions of the simulated scenario take place in an epithelium tract and in the surrounding lymph nodes. Hence this is a good approximation and gives to the model implementation improvements in both speed and simplicity.

As all the entities and the interactions have been identified and conceptually introduced, one has to deal with the final step of the modeling process i.e., the choice of the more suitable modeling technique.

Using our previous experiences in the field of simulation of different pathologies and the related immune system response [16]–[18], [24]–[28], we choose to adopt an agent based modeling (ABM) approach as a computational framework.

Although ABM is not subject to a deep analytical analysis by presently known methods, it has many advantages.

The ABM is stochastic, so it is possible to estimate the distribution of behaviors exhibited by the entire system, not just the average, in fact determinism is avoided and it is possible to take into account the effect of the spatial distribution easily.

The ABM is also able to accurately represent many of the biological processes of interest so that the approximations in the model are usually more biological in character than mathematical.

Moreover it is possible to approaching nonlinearities in as simple way because they are not intrinsically hard to handle. This also means that it is possible to add complexity or modify the model without introducing any new difficulties in solving it.

Most mathematical models of the immune response are deterministic and use, for example, differential equations (solved by numerical integration) to model the biological interactions among cells and molecules. However, the assumptions made by the deterministic method do not hold for many processes that are sensitive to the behavior of a relatively small number of entities.

Another problem of equations is given by their formalism. The “language” used by equations is not usually known to biologists, making the communication with mathematicians/Computer scientists even more hard and damaging the interdisciplinary collaboration for any model to be built [23].

This method is also intrinsically numerically stable thanks to the fact that most of the variables representing the attributes of the entities are integers and very few floating point operations are required. Modeling the immune system response with a differential equation model, trying to represent all the immunological aspects modeled by the ABM, is discouraging. To justify this statement consider that to coherently model the immune system it is fundamental to take into account entities receptors variability. Receptors variability can reach approximately 10^7^ different receptors at the same time in the host (from a range of 10^13^ − 10^15^ possible receptors) and, even modeling a smaller receptor range (i.e. 10^3^ − 10^6^), it can represent a problem.

To model, for example, the clonal selection process problem with an ODE, one should take into account a number of equations that is of the same order of the allowed receptors variability. Moreover presentation or stimulation processes involve different types of specialized cells, so the number of equations grows further.

Imagine a typical process of the immune system, i.e. when an cell A, after an interaction with a cell B, switches its internal state and mutates into a new cell C (for example a B lymphocyte that differentiates into a plasma cell after stimulation by a TH lymphocyte).

Let R be the allowed set of possible receptors, A_i_ be the set of entities of type A with receptor i ∈ R and C_i_ be the set of entities of type C with receptor i ∈ R (even if affinity maturation occurs in real immune system, we suppose for simplicity that the receptor does not change during the switch).

The A_i_ cells can hypothetically interact with all B_j_ , j  =  1...|R|, and the stimulation depends by the affinity between receptors i and j, so one has:
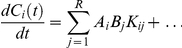
where k_ij_ represents the affinity ratio between the receptors i and j and i, j  =  {1, . . . , |R|}. This differential equation has to be repeated for all the A clones (one for each different receptor i, i ∈ R).

If the biological problem is not homogeneous in space, the use of a density function f (t, x) should be required for every population (i.e. a partial differential equations system), further increasing the complexity of the model.

Moreover immune system interactions and events are not deterministic. This means that one should include a source of randomness into the equations. Stochastic differential equations (SDE) or stochastic partial differential equations (SPDE) should be then used.

Summarizing, if R is allowed receptor variability (i.e. 2^12^ in SimB16) and S the number of different kinds of entities with specialized receptors (i.e. S  =  3 for B, TC and TH representation, forgetting about plasmacells, antibodies, antigens and entities state-changes), a system of (at least) R · S ( =  3 · 2^12^ ≈ 10^4^) stochastic partial differential equations (with various non-linearities) has to be used to tackle the biological problem in a similar way the CS automaton does.

### Model implementation: SimB16 simulator

The computer implementation of the model (SimB16 hereafter) has three main classes of parameters: *i)* values known from standard immunology literature; *ii)* parameters strictly correlated with the specific biological scenario we want to simulate, i.e. parameters that measure the B16-OVA melanoma dynamics, its behavior and its interactions with the immune system of the host; *iii)* parameters with unknown values which we set to plausible values after performing a series of systematic tests.

Table 1 details the values of the parameters retrieved from the literature. Table 2 shows specific parameters of B16-OVA melanoma.

**Table 1:** SimB16 parameters with known values retrieved from immune system specific literature.

Entity	Initial quantity (per µL)	Half life
B	260	3.3 days
TH	200	3.3 days
TC	434	3.3 days
cDC	351	3.3 days
M	351	3.3 days
EP	351	3.3 days
NK	351	3.3 days
P	0	3.3 days
IC	0	4.0 days
IL-2	0	1.6 days
CA	0	1.6 days
IgG	0	23.0 days

CA stands for Chemo-Attractans.

**Table 2:** SimB16 parameters with known values retrieved from specific literature.

Entity	Initial quantity (per µL)	Half life
B16 cancer cells	0	10 years
Anti-CD137	N/A	23 days
OT-1 T-cells	N/A	3.3 days

N/A means not applicable.

SimB16 has set of free parameters that can be used to tune the model results with experimental data. The list of these parameters and their final used values are quoted in Table 3. Tuning parameters were initially set using previous modeling experience and then adjusted to reproduce experimental behavior. The first parameter we set is *nbit_str* that determines the repertoire size. It indicates the number of bits used to represent the molecules and the cells binding sites like cell receptors and antigen peptides and epitopes. It was set to 12 corresponding to a potential repertoire of 4096 cell receptors. This is obviously very poor respect to the real immunological repertoire, but it was sufficient to capture the global behavior of the B16-OVA/immunotherapy process. The parameter *min_match* specifies the minimal number of matching bits that are required to have a non-zero probability to bind; *affinity_level* is the probability to interact between two binding sites whose match is min_match; *max_lfact* regulates the probability for a cell that is duplicating to create a new cell; *IL2_eff* is a factor expressing the efficiency of interleukin-2 in stimulating growth of the lynphocytes; *thymus_eff* represents the efficiency of the thymus in selecting non self-reactive thymocytes. In general the fraction of circulating auto-reactive TH cells should be below 0.1%.

**Table 3:** SimB16 tuning parameters.

Parameter	Value
Timestep	8 hours
hyper_mut	10^−4^ in 8 hours
plasma_rel	10 ng/µ per 8 hours
prob_M_Ag	10^−2^ in 8 hours
prob_M_IC	10^−1^ in 8 hours
prob_cDC_Ag	2×10^−2^ in 8 hours
B_dup	16
TH_dup	16
TC_dup	16
nbit_str	12
min_match	9
affinity_level	5×10^−2^
max_lfact	5
IL2_eff	100%
thym_eff	99.9%

In order to catch the evolution of all quantities, the time step of a simulation must be lesser than all characteristics times. We chose a time step of 8 hours to satisfy this requirement.

Parameter *hyper_mut* is the per-bit mutation probability for the antibodies. The hyper-mutation rate of antibodies is taken as the suggested value in Celada, 1996; *plasma_rel* controls the quantity of specific IgG antibodies released by a plasma B cell per time step; *prob_M_Ag* is the probability for a macrophage to phagocyte/internalize an antigen; *prob_M_IC* is the probability for a macrophage to phagocyte an immune complex; *prob_cDC_Ag* is the probability for a dendritic cell to phagocyte/internalize an antigen. This was set to a higher level than the *prob_M_Ag* as DC are known to be better antigen presenting cells; *B_dup* is the number of time steps a B cells creates a copy of itself when duplicating; *TH_dup* is the number of time steps a TH cells creates a copy of itself when duplicating; *TC_dup* is the number of time steps a TH cells creates a copy of itself when duplicating.

With these settings, the model showed a good robustness, giving reasonable output. This means that if we slightly change parameters such as the initial leukocyte formula, the half life of entities, and so on, the model consistently varies its results, without biological discrepancy when compared with available experimental data retrieved from the above cited literature.

SimB16 takes care of the main immune system functions and peculiar characteristics, such as diversity of specific elements, major histocompatibility classes restriction, clonal selection by antigen affinity, thymus education of T cells, antigen processing and presentation (both the cytosolic and endocytic pathways are implemented), cell-cell cooperation, homeostasis of cells created by the bone marrow, hypermutation of antibodies, cellular and humoral response and immune memory. Receptors, ligands and immune system specificity are implemented in SimB16 by a *bit-string polyclonal lattice method*. This was well-described method and the interested reader can found additional information in specific literature [19].

Immune system entities interact both each other and with the cells and molecules of the body. From the point of view of biology, an interaction is a complex event that includes chemical, biological and dynamical actions. We implemented both recognition phase and affinity eventually enhanced by adjuvants. When two entities, which may interact, lie in the same lattice site then they interact with a probabilistic law. All entities that may interact and are in the same site have a positive interaction.

Physical proximity is modeled through the concept of lattice-site. All interactions among cells and molecules take place within a lattice-site in a single time step, so that there is no correlation between entities residing on different sites at a fixed time.

The simulation space is represented as an *L*×*L* hexagonal (or triangular) lattice (six neighbors), with periodic boundary conditions to the left and right side, while the top and bottom are represented by rigid walls. All entities are allowed to move with uniform probability between neighboring lattices in the grid with equal diffusion coefficient. We considered 3600 mm^2^ (60 mm×60 mm) of mouse tissue that we simulated using a lattice grid size of 946×946.

Compared to the complexity of the real biological scenario, the model can be extended in many aspects. On the other hand the model is complete enough and is able to describe the major aspects of the modeled experiment.

In order to model the melanoma growth pattern, we followed the procedure described hereinafter. The first step to accomplish is to keep track of the temporal evolution of the cancer in absence of any treatment. To this end the diameter of melanomas for untreated mice has been measured and stored at different times during the experiment.

Supposing a disk-shaped layout for the melanoma and having knowledge of the mean diameter of melanoma cancer cells, we estimated the number of cancer cells (in the observed melanomas) for all the measurements made.

This data has been used with a curve fitting procedure to estimate the unknown parameters needed to model the cancer growth kinetics under the hypothesis that the melanoma growth followed a Gompertz law[20].

The law, which is commonly considered suitable for describing populations growths, uses two factors: a growth factor that decreases in time and a constant mortality factor. Thanks to the growth factor deceleration, the dimension of the population tends asymptotically to a certain threshold (carrying capacity) giving a sigmoid shape to the law.

Once the unknowns of the growth law have been determined, they can be used to reproduce the growth kinetics in time of the melanoma. In particular, we approximate the differential form of the Gompertz law with the forward Euler discretization method, obtaining:
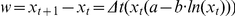
where *x_t_* and *x_t+1_* are the number of cancer cells at time *t* and *t+1*, *a* and *b* are the fitted unknowns of the growth law, and Δ*t = 1* since the simulator uses discrete time-steps. Starting from time-step 0, where it is supposed *x_0_* = 200000 (we suppose that nearly 60% of the injected cancer cells die before to settle and duplicate), the model determines at each time-step *t* the expected number of newborn cancer cells *w* (* = x_t+1_*− *x_t_*) that will be introduced into the simulation at time-step *t+1* on the basis of the actual number of cancer cells *x_t_*.

Chemotaxis is a biological phenomenon in which some cells, like immune system cells, direct their movements according to certain chemical signals. Chemotaxis is a very complex process involving many factors such as short and long range interactions and no model is probably able to completely represent this phenomenon, since it has not yet fully understood[21].

In a first attempt to mimic short range chemotaxis effects, higher probabilities of being chosen are given to sites containing chemoattractans released by endothelial cells, activated macrophages and activated OT-1 cells.

### In vivo experimental settings

#### Immunotherapy setting

B16 melanoma cell line was derived from an aggressive spontaneous melanoma in pure C57BL6 and B16F10 was derived as a clonal variant from a lung metastasis of this cell line. B16-OVA was transduced with the chicken ovalbumin gene to use it as a model tumor antigen. In tumor immunology these variants are considered poorly immunogenic in the sense that immune-mediated rejections or growth retardations are difficult to achieve.

The experimental setup is oriented to model therapeutic synergy between anti-CD137 monoclonal antibodies and adoptive T cell therapy in melanoma. B16-OVA is a poorly immunogenic murine tumor that does respond to neither anti-CD137 mAb nor adoptive transfer used as monotherapy. The treatment protocol includes a single injection of anti-CD137 mAb and adoptive T cell transfer of OVA specific TCR-transgenic CD8 T cells.

#### Cell lines

B16-OVA was obtained and authenticated from ATCC. Cells were cultured in complete RPMI medium (RPMI 1640 with Glutamax [Gibco, Invitrogen, CA] containing 10% heat-inactivated FBS (SIGMA-ALDRICH, UK), 100 IU/ml penicillin and 100μg/ml streptomycin (Biowhittaker, Walkersville, MD) and 50 µΜ 2-mercaptoethanol.[Gibco]). B16-OVA was cultured under G418 selection (1 mg/mL, Gibco).

#### Mice

C57 BL/6 female mice (6–8 weeks old) were purchased from Harlan Laboratories (Barcelona, Spain). OT-I TCR-transgenic mice were from The Jackson Laboratory (Barcelona, Spain) and bred in our animal facility under specific pathogen-free conditions. Animal procedures were conducted under institutional guidelines that comply with national laws and policies (study 066/10).

#### Antibodies

Anti-CD137 mAb is produced from the 2A hybridoma, kindly provided by Dr. Lieping Chen. The mAb produced by this hybridoma was purified from culture supernatant by affinity chromatography in sepharose protein G columns (GE Healthcare Bio-sciences AB, Uppsala, Sweden) dialized and quality controlled including determinations of lipopolysaccharide (LPS) concentration (Antibody BCN, Barcelona, Spain, as a contractor). Control IgG from rat serum was obtained from Sigma-Aldrich.

#### In vivo tumor growth and treatment

0.5×10^6^ B16-OVA cells were injected in 100 mL PBS subcutaneously in the right flank of C57 BL/6 mice. OT-1 T cells were prepared from the spleens of OT-I TCR transgenic mice and activated with 5 ug/mL of OVA257-264 peptide (NeoMPS, Strasbourg, France) for 48 hours at 37°C in 5% CO_2_ and washed extensively before i.v. inoculation. On day 3 or 8 after tumor injection mice received i.p. 100 ug of anti-CD137 monoclonal antibody or control rat IgG and 2×106 activated or naive OT-1T cells i.v. Tumor growth was evaluated by measuring 2 perpendicular diameters with a digital caliper every 3–4 days. Mice were sacrificed when tumor sizes reached 450 mm^2^.

### Ethics

The protocol was approved by Comite de Etica para la Investigacion Animal of the University of Navarra (ID:066/10).

## References

[1] Morgan RA, Dudley ME, Wunderlich JR, Hughes MS, Yang JC (2006). Cancer regression in patients after transfer of genetically engineered lymphocytes.. Science.

[2] Rosenberg SA, Restifo NP, Yang JC, Morgan RA, Dudley ME (2008). Adoptive cell transfer: a clinical path to effective cancer immunotherapy.. Nat Rev Cancer.

[3] Melero I, Martinez-Forero I, Dubrot J, Suarez N, Palazon A (2009). Palettes of Vaccines and Immunostimulatory Monoclonal Antibodies for Combination.. Clin Cancer Res.

[4] Perez-Gracia JL, Berraondo P, Martinez-Forero I, Alfaro C, Suarez N (2009). Clinical development of combination strategies in immunotherapy: are we ready for more than one investigational product in an early clinical trial?. Immunotherapy.

[5] Tirapu I, Arina A, Mazzolini G, Duarte M, Alfaro C (2004). Improving efficacy of interleukin-12-transfected dendritic cells injected into murine colon cancer with anti-CD137 monoclonal antibodies and alloantigens.. Int J Cancer.

[6] Tirapu I, Huarte E, Guiducci C, Arina A, Zaratiegui M (2006). Low surface expression of B7-1 (CD80) is an immunoescape mechanism of colon carcinoma.. Cancer Res.

[7] Palazon A, Teijeira A, Martinez-Forero I, Hervas-Stubbs S, Roncal C (2011). Agonist anti-CD137 mAb act on tumor endothelial cells to enhance recruitment of activated T lymphocytes.. Cancer Res.

[8] Broll K, Richter G, Pauly S, Hofstaedter F, Schwarz H (2001). CD137 expression in tumor vessel walls. High correlation with malignant tumors.. Am J Clin Pathol.

[9] Melero I, Murillo O, Dubrot J, Hervas-Stubbs S, Perez-Gracia JL (2008). Multi-layered action mechanisms of CD137 (4-1BB)-targeted immunotherapies.. Trends Pharmacol Sci.

[10] Melero I, Shuford WW, Newby SA, Aruffo A, Ledbetter JA (1997). Monoclonal antibodies against the 4-1BB T-cell activation molecule eradicate established tumors.. Nat Med.

[11] Melero I, Hervas-Stubbs S, Glennie M, Pardoll DM, Chen L (2007). Immunostimulatory monoclonal antibodies for cancer therapy.. Nat Rev Cancer.

[12] Ito F, Li Q, Shreiner AB, Okuyama R, Jure-Kunkel MN (2004). Anti-CD137 monoclonal antibody administration augments the antitumor efficacy of dendritic cell-based vaccines.. Cancer Res.

[13] May KF, Chen L, Zheng P, Liu Y (2002). Anti-4-1BB monoclonal antibody enhances rejection of large tumor burden by promoting survival but not clonal expansion of tumor-specific CD8+ T cells.. Cancer Res.

[14] Dubrot J, Milheiro F, Alfaro C, Palazon A, Martinez-Forero I (2010). Treatment with anti-CD137 mAbs causes intense accumulations of liver T cells without selective antitumor immunotherapeutic effects in this organ.. Cancer Immunol Immunother.

[15] Melief CJ (2008). Cancer immunotherapy by dendritic cells.. Immunity.

[16] Pappalardo F, Pennisi M, Castiglione F, Motta S (2010). Vaccine protocols optimization: in silico experiences.. Biotechnol Adv.

[17] Pappalardo F, Musumeci S, Motta S (2008). Modeling immune system control of atherogenesis.. Bioinformatics.

[18] Castiglione F, Pappalardo F, Bernaschi M, Motta S (2007). Optimization of HAART with genetic algorithms and agent-based models of HIV infection.. Bioinformatics.

[19] Motta S, Castiglione F, Lollini P, Pappalardo F (2005). Modelling vaccination schedules for a cancer immunoprevention vaccine.. Immunome Res.

[20] Palladini A, Nicoletti G, Pappalardo F, Murgo A, Grosso V (2010). In silico modeling and in vivo efficacy of cancer-preventive vaccinations.. Cancer Res.

[21] Baldazzi V, Paci P, Bernaschi M, Castiglione F (2009). Modeling lymphocyte homing and encounters in lymph nodes.. BMC Bioinformatics.

[22] Olofsson PS, Soderstrom LA, Wagsater D, Sheikine Y, Ocaya P (2008). CD137 is expressed in human atherosclerosis and promotes development of plaque inflammation in hypercholesterolemic mice.. Circulation.

[23] Pappalardo F, Lefranc M, Lollini PL, Motta S (2010). A novel paradigm for cell and molecule interaction ontology: from the CMM model to IMGT-ONTOLOGY.. Immunome Research,.

[24] Pappalardo F, Mastriani E, Lollini PL, Motta S (2005). Genetic Algorithm Against Cancer.. Lecture Notes in Artificial Intelligence.

[25] Pennisi M, Catanuto R, Pappalardo F, Motta S (2008). Optimal vaccination schedules using simulated annealing.. Bioinformatics.

[26] Pappalardo F, Halling-Brown M, Rapin N, Zhang P, Alemani D (2009). ImmunoGrid, an integrative environment for large-scale simulation of the immune system for vaccine discovery, design and optimization.. Briefings in Bioinformatics.

[27] Halling-Brown M, Pappalardo F, Rapin N, Zhang P, Alemani D (2010). ImmunoGrid: towards agent-based simulations of the human immune system at a natural scale.. Philos T R Soc A.

[28] Pennisi M, Pappalardo F, Palladini A, Nicoletti G, Nanni P (2010). Modeling the competition between lung metastases and the immune system using agents.. BMC Bioinformatics.

